# Predictors of spiritual care provision for patients with dementia at the end of life as perceived by physicians: a prospective study

**DOI:** 10.1186/1472-684X-13-61

**Published:** 2014-12-19

**Authors:** Jenny T van der Steen, Marie-José HE Gijsberts, Cees MPM Hertogh, Luc Deliens

**Affiliations:** Department of General Practice & Elderly Care Medicine, EMGO Institute for Health and Care Research, VU University Medical Center, van der Boechorststraat 7, 1081 BT Amsterdam, the Netherlands; Department of Public and Occupational Health, EMGO Institute for Health and Care Research, VU University Medical Center, van der Boechorststraat 7, 1081 BT Amsterdam, the Netherlands; End-of-Life Care Research Group, Vrije Universiteit Brussel & Ghent University, Laarbeeklaan 103, 1090 Brussels, Belgium

**Keywords:** Dementia, Spirituality, Palliative care, Nursing homes

## Abstract

**Background:**

Spiritual caregiving is part of palliative care and may contribute to well being at the end of life. However, it is a neglected area in the care and treatment of patients with dementia. We aimed to examine predictors of the provision of spiritual end-of-life care in dementia as perceived by physicians coordinating the care.

**Methods:**

We used data of the Dutch End of Life in Dementia study (DEOLD; 2007–2011), in which data were collected prospectively in 28 Dutch long-term care facilities. We enrolled newly admitted residents with dementia who died during the course of data collection, their families, and physicians. The outcome of Generalized Estimating Equations (GEE) regression analyses was whether spiritual care was provided shortly before death as perceived by the on-staff elderly care physician who was responsible for end-of-life care (last sacraments or rites or other spiritual care provided by a spiritual counselor or staff). Potential predictors were indicators of high-quality, person-centered, and palliative care, demographics, and some other factors supported by the literature. Resident-level potential predictors such as satisfaction with the physician’s communication were measured 8 weeks after admission (baseline, by families and physicians), physician-level factors such as the physician’s religious background midway through the study, and facility-level factors such as a palliative care unit applied throughout data collection.

**Results:**

According to the physicians, spiritual end-of-life care was provided shortly before death to 20.8% (43/207) of the residents. Independent predictors of spiritual end-of-life care were: families’ satisfaction with physicians’ communication at baseline (OR 1.6, CI 1.0; 2.5 per point on 0–3 scale), and faith or spirituality very important to resident whether (OR 19, CI 5.6; 63) or not (OR 15, CI 5.1; 47) of importance to the physician. Further, female family caregiving was an independent predictor (OR 2.7, CI 1.1; 6.6).

**Conclusions:**

Palliative care indicators were not predictive of spiritual end-of-life care; palliative care in dementia may need better defining and implementation in practice. Physician-family communication upon admission may be important to optimize spiritual caregiving at the end of life.

## Background

Spiritual caregiving may contribute to wellbeing at the end of life, as shown in palliative populations of mostly cancer patients [1–4]. Spiritual caregiving in dementia may be a neglected area, with little research available [5–7]. For example, in a UK hospital, religious beliefs of dementia patients were less frequently documented than in patients without dementia [8]. Similarly, in a US nursing home setting, cognitively impaired residents less frequently received support or care by facility staff for spiritual needs [9].

In dementia and at the end of life, spiritual caregiving poses particular challenges. For example, it may be difficult to predict the end of life, and to communicate verbally due to cognitive impairment, perhaps with superimposed acute illness [5]. Conceptually, cognitive appraisal is part of some definitions of spirituality at the end of life [10, 11]. However, rituals and music may be recognized even in severe dementia [7, 12–14]. Further, recent reviews indicate that there is some evidence of beneficial effects, also in dementia, of spiritual interventions and spirituality and religiousness on, for example, coping, wellbeing, and behavior [6, 7].

We do not know when patients with dementia do or do not receive spiritual end-of-life care. In long-term care settings, the provision of spiritual care has been associated with families’ perception of better overall care in the last month of life [9]. This retrospective work suggests that families appreciate spiritual end-of-life care, or, that a high quality of care standard promotes spiritual caregiving at the end of life. In addition to high-quality care, the related and overlapping notions of person-centered and palliative care may promote spiritual caregiving. That is, spiritual care is part of palliative care [15]. Further, at the individual level spiritual care should be consistent with, so may be related to patient- and family-centered principles [5, 16]. It should therefore consider the individual spiritual background, and respect any sensitivities. An individualised approach is particularly important in western, secularized countries where physicians are often less spiritual or religious than their older patients.

However, prospective studies with a clear temporal relationship relating such variables to provision of spiritual end-of-life care later are lacking. To our best knowledge, no study has systematically reviewed potential predictors of the provision of spiritual end-of-life care. Further, spirituality is an important theme in the nursing literature, but less is known about physicians’ perceptions of spiritual caregiving, even though they are part of the team or have an important role in the provision of palliative care at the end of life, which includes spiritual caregiving. To explore how to support the physician’s role in the spiritual caregiving at the end of life, we examine potential predictors of the provision of spiritual end-of-life care as perceived by physicians in a cohort of newly admitted residents with dementia in the long-term care setting of the secularized country of the Netherlands.

## Methods

### Design

Data were obtained from the Dutch End of Life in Dementia (DEOLD) study which involved both prospective data collection in 28 long-term care facilities and retrospective (after death only) data collection in 6 facilities with survival assessed up to summer 2011. The study’s design is detailed elsewhere [17], and potential predictors for the secondary analyses to address our research question are described in detail in Table 1. We used the data collected prospectively regarding a cohort of newly admitted residents; the assessments were performed between January 2007 and July 2010. The study protocol was approved by the Medical Ethics Review Committee of the VU University Medical Center. Families of residents enrolled in the study provided written informed consent to participate in the study shortly after the resident’s admission [17].

**Table 1:** **Potential predictors of the provision of spiritual end-of-life care through previous work, and definitions**

Potential predictor (4 categories of which 3 indicate specific concepts)	Justification of possible predictive properties and expected association through previous work	Operationalization	
		Variable and measurement level*	Definitions of variable and response options, missing data
**(1) Quality of care**			
Long-term care facility type/physician presence	Dutch and US physicians who are more present are more certain of family preferences [18]. Further, better quality of end-of-life care was reported in Dutch nursing homes compared to residential homes [19].	Nursing vs. residential home	*Definition:* Dutch nursing homes have elderly care physicians on the staff, and outreach to units for dementia in residential homes of the same organization with no continuous physician presence.
		Facility	*Time frame*: Unchanged throughout data collection; for residents who moved: refers to location after move.
			*Perspective:* Coordinating physician and if missing, assessed through the facility’s website.
			*Missing data*: No missing values.
Urbanization level	Better overall quality of care was provided in less urbanized areas according to some reports on nursing home care in the Netherlands (references in Dutch provided elsewhere [19]).	Located in town vs. large city	*Definition*: Facility located in small city, town, village, or rural area versus in one of the four largest cities, all in the western part of the Netherlands.
	It should be noted that secularization may be prominent in urbanized areas, which suggests it might also relate to to spiritual caregiving in other ways.	Facility	*Time frame*: Unchanged throughout data collection; for residents who moved: refers to location after move.
			*Missing data*: No missing values.
Staffing	Quality of care was lower with nursing staff shortage and higher turnover [20–23]	Enough nursing staff	*Definition*: Sufficiency of nursing staff as perceived by the coordinating physician
		Facility	*Time frame*: Conclusion of data collection.
			*Perspective*: The coordinating physician.
			*Response options*: More than enough, just enough (combined), versus not enough.
			*Missing data*: Loss to follow up- for those who moved to another facility (6 cases) was coded as missing.
Evaluation of quality of care - overall	An association of spiritual caregiving with family satisfaction with end-of-life care has been reported in a US study [9].	Satisfaction with care	*Definition*: Perception or satisfaction of care measured with the End-of-Life in Dementia-Satisfaction With Care (EOLD-SWC) scale [24]. It represents quality of care as perceived by families [25].
		Resident	*Time frame*: We used the baseline assessment^†^ which referred to the first 8 weeks after admission. The EOLD-SWC has been used for timeframes other than the last period before death in other prospective work as well [26].
			*Perspective*: Family.
			*Response options*: 10 4-point items are summed and total scores range 10–40, with higher scores representing better quality of care.
			*Missing data*: Missing values (40) include non-random missing for those who died before the baseline assessment.
Evaluation of quality of care – communication specifically	Communication with families may be specifically important for the physician to optimally coordinate care, including spiritual care. Further, communication is a major aspect of quality of end-of-life care and families’ evaluation – i.e., satisfaction with end-of-life care including “timeliness of information, counseling” and “interpersonal and communication style” is an important outcome on its own [27].	Satisfaction with communication	*Definition*: Item: “Are you satisfied with how the communication with the physician(s) is going (discussions on future care, goals of treatment, and current care)?”
		Resident	*Time frame*: Baseline.^†^
			*Perspective*: Family.
			*Response options*: We created a 0–3 satisfaction scale with the response options: “satisfied in every respect” (3), “satisfied about the main elements” (2), “neutral” (1), “not satisfied” (0), “did not talk to physician(s) yet, while I would have wanted to (0), did not talk to physician(s) yet and I do not think that is needed yet (1).
			*Missing data*: Missing values (37) include non-random missing for those who died before the baseline assessment.
**(2) A more individualised or more person-centered approach of care; religious backgrounds**			
Philosophy of care related to individualised approach	Individualised person-centered approach: home-like, small-scale living might involve a more individualised approach. The literature on studies performed in the Netherlands reports it possibly relates to better quality of life although unclear how it relates exactly to quality of care [28, 29].	Small-scale living	*Definition:* Small-scale living arrangement for dementia available.
		Facility/resident	*Response options*: At the facility level (descriptive; patient-level data used for analyses): all of the residents the facility enrolled in the study; some of the residents; no small-scale living for dementia available.
			*Time frame*: Assessed at the conclusion of the study for the period of data collection, and any changes during that period.
			*Missing data:* 1 case.
Religious affiliation	In a US study, religiously-affiliated facilities were comparable to nonaffiliated facilities in providing on-site religious services, but more likely to provide individual counseling by clergy or chaplains [9]. Therefore, a more individualised approach to spiritual caregiving may be assumed. US nursing homes with a religious affiliation were more likely to provide spiritual end-of-life care to their residents [30]. Further, nursing homes with a strong religious affiliation also provided better end-of-life care in a previous Dutch study [31], and more religion-oriented homes might also adhere to a palliative care approach more strongly.	Strong religious affiliation	*Definition:* Strong, explicit religious affiliation in place versus no affiliation or only historically.
		Facility	*Timeframe*: Unchanged throughout data collection; for residents who moved: refers to location after move.
			*Perspective*: Assessed by coordinating physician in discussions with researcher.
			*Missing data*: No missing values.
Religious backgrounds and concordance care provider - patient	Families and physicians with any specific background may be more attentive to an individual’s spiritual needs. An individualised person-centered approach is indicated by spiritual care more frequently being provided to residents with a specific religious background in particular when the physician does not have a specific background. That is, providing spiritual care when physician and patient have the same spiritual background does not need a special individualised approach, but it is indicative of such approach if spiritual care is being provided despite dissimilar spiritual backgrounds.	Religious background	*Definition:* Any specific religious background.
		Physician, resident	*Response options*: We combined any specific religious background (“Protestant”, “Catholic”, “Muslim”, “Humanist”, “Jewish”, and “other”) versus “no specific religious background” for physicians (self-report), and families and residents (family report). We also created a variable that compared such background of the physician and the resident.
			*Time frame*: Residents and families: baseline assessment.^†^ For families, we used the religion of the family who completed the baseline assessment. Physician’s religious background was assessed midway study.
			*Missing data:* 21 physician responses, 12 for residents, and 13 for families. Resident-physician combined: 32 missing values.
Importance of faith or spirituality in life and concordance care provider - patient	An individualised person-centered approach is indicated by spiritual care more frequently being provided to residents for whom faith or spirituality was important in life, as found in a US study [30], and in particular when the physician does not find it important for him- or herself.	Importance of faith or spirituality	*Definition*: Item: “How important is (resident: was) faith or spirituality in your life (resident: to your family/loved one)?”
		Physician, resident	*Response options*: We tested “very important” versus “somewhat important”, “not at all important”, and “don’t know” because there was not always a stepwise increase for the three hierarchical levels, and the distributions did not always allow for analyzing the full categorical variables with a reference category. We also created a variable that compared the physician’s and the resident’s faith or spirituality being very important.
			*Time frame*: Same as religious background.
			*Perspective*: Physicians (self-report), families and residents (family report).
			*Missing data*: Same as religious background.
Religious activities involvement	An individualised person-centered approach is indicated by spiritual care more frequently being provided to residents who used to attend religious serves more frequently. It parallels the outcome which also refers to formal and visible spiritual care provision, including explicit reference to rituals.	Frequency of attending religious services	*Definition*: Item: “How often do you attend church or other religious services?”
		Physician, resident	*Response options*: “More than once a week”, “every week”, “two or three times a month”, “once a month or so”, “once or twice a year”, “never”, and, for families only, regarding residents and themselves, “don’t know”. We transformed the responses into a 0–5 scale, recoding don’t know as missing and after confirming there was a stepwise increase in the association with the outcome.
			*Time frame*: Same as religious background.
			*Perspective*: Physicians (self-report), families and residents (family report).
			*Missing data*: 21 physician responses, 13 for residents, and 14 for families.
Quality of family-physician relationship	Assuming that trust is built up when relationships develop favorably, it may indicate a more individualised approach.	Family trust	*Definition*: Item: “How much trust do you put in that the physician involved in care for your family/loved one tries hard to make the best of it for your family/loved one?
		Resident	*Response options:* We created a 1–5 scale with the response options “a very large amount of trust (5)”, “a great deal (large amount) of trust (4)”, “somewhat trust (3)”, “little trust (2)”, and “very little trust (1)”.
			*Time frame*: Baseline assessment.^†^
			*Perspective*: Families.
			*Missing data*: Missing values (37) included non-random missing for those who died before the baseline assessment.
**(3) Palliative care**			
Palliative care explicitly provided at location	A positive spill-over effect of US hospice services on hospitalization rates of nursing home residents who were not on hospice has been noted by Miller et al. [32] who suggested this was possibly through diffusion of palliative care philosophy and practices. Further, a US study found residents of nursing homes with a hospice unit or providing hospice services more likely to have received spiritual end-of-life care [30].	Palliative care unit	*Definition*: Palliative care unit (not commonly used for dementia patients) available in the facility vs. not available.
		Facility	*Time frame*: At start of data collection, and confirmed unchanged midway and at conclusion of data collection.
			*Perspective*: Coordinating physician.
			*Missing data*: 6 cases due to move to non-participating facilities.
Palliation as the care goal that takes priority	Different care goals may coexist, but palliative care may be compatible with prioritizing comfort and maintaining function [5].	Comfort goal of care	*Definition*: The care goal that takes priority. A comfort goal combines “palliative” and “symptomatic” with explanation that both are aimed at wellbeing and quality of life with only for a symptomatic additional prolonging of life being undesirable [33], versus “life prolongation”, “maintaining or improving of functioning”, “other”, or “no global care goal assessed yet”. We did not include functioning for a better distribution.
		Resident	
			*Time frame*: Baseline,^†^ after the care planning meeting which Dutch law requires within 6 weeks from admission [34, 35].
			*Perspective*: Physician.
			*Missing data*: Missing values (37) included non-random missing for those who died before the baseline assessment.
Anticipating death	Palliative care explicitly refers to dying as a normal process, and the prevention of suffering by means of early identification [15].	Death expected	*Definition:* Item: “If you think back to one month before your family/loved one died, do you feel like at that time you expected that he/she was going to die?”
			*Time frame*: After-death assessment.^†^
	Further, quality of end-of-life care may be better when death is expected, with more opportunities to arrange the care the resident needs, and ensure a comfortable death [36].	Resident	*Perspective*: Family.
			*Response options*: “Yes”, “no”, “don’t know”. For analyses, we combined the last two options.
			*Missing data*: Missing values (31) included non-random missing values for those who died before the baseline assessment.
Recognizing terminality	Recognizing dementia as a terminal disease may be a basis for the provision of palliative care. In the DEOLD study, when families believed dementia was a disease you can die from, the resident had a more comfortable death [34]. It may therefore also indicate better quality of care.	Perception of dementia as a disease you can die from	*Definition:* Item: “In your opinion, dementia is a disease you can die from”.
		Resident	*Time frame*: Baseline assessment^†^
			*Perspective*: Family.
			*Response options*: “Completely disagree”, “partly disagree”, “neither agree, nor disagree”, “partly agree”, “completely agree” and “do not know”. We used a 1–5 agreement scale combining “don’t know” and “neither agree, nor disagree”. [34].
			*Missing data*: Missing values (38) included non-random missing values for those who died before the baseline assessment.
**(4) Other factors or unclear expectation with regard to the direction of a possible association**			
Facility size and type	The literature reports associations with quality of care in opposite directions; references are provided elsewhere (online Annex [17]).	Number of beds	*Definition*: Number of psychogeriatric (dementia) care beds in the facility.
	Residents of small US residential homes/assisted living facilities (< 16 beds) were less likely to receive spiritual end-of-life care [30].	Facility	*Timeframe*: If changed during data collection, we calculated the mean number of beds over assessments at the start, mid-way and conclusion of the data collection period.
			*Missing data*: No.
Demo-graphics	A US study found no significant association with resident gender or age in unadjusted (univariable) analyses [9]. However, demographics may relate to religiousness.	Gender and age	*Definition and perspective*: Gender and age of physician (physician report) and of family and resident (family report). We report on the physician involved in end-of-life care, and the family involved at baseline.
		Physician, resident	*Timeframe*: All refer to the age when the resident died.
			*Missing data*: 12 for physicians, 0 for residents, and for families, 2 missing gender and 12 missing age.
Dementia severity	Less severe dementia may be associated with more frequent spiritual care in parallel with less frequent care compared to patients without dementia [8, 9].	Dementia severity	*Definition:* Bedford Alzheimer Nursing Severity-Scale (BANS-S) score, range 7–28 [37]. Scores of 17 and higher represent severe dementia [38].
		Resident	*Timeframe*: Baseline.^†^
			*Perspective*: Physician (this item was completed by the nurse supervised by the physician in 68.9% of cases).
			*Missing data*: 4 missing values.
Closeness of relationship	Individualised approach yet not attributable to professional caregivers. Spouses and children may be more cognizant regarding the resident’s spiritual needs and background compared with other informal caregivers.	Relationship	*Definition and response options:* Relationship with resident of family involved at baseline: “spouse” combined with “partner;” “child;” and “other” which combined “grandchild”, “sibling”, “niece/nephew”, “legal guardian, and “other”.
		Resident	*Timeframe*: Baseline.^†^
			*Perspective*: Family.
			*Missing data*: 12 missing values.

^*^Family and resident level are the same, because families provided a single after-death assessment on their deceased relative.

^†^Time frame: “baseline” refers to a resident-level assessment eight weeks after admission to the facility, “after death” was around two months after death for family, and within two weeks after death for physicians.

The main purpose of the DEOLD study was to assess factors associated with after-death patient outcomes. Inclusion criteria were newly admitted to a “psychogeriatric” ward/unit (almost all dementia) of a nursing home, or a residential home facility covered by elderly care physician services, a physician’s diagnosis of dementia, admitted for long-term care, and having a family representative able to understand and write Dutch or English.

Physician and family caregiver assessments were conducted eight weeks after admission to the facility (baseline), semi-annually, and after death (around two months after death for family, and within two weeks for physicians; see also “Time frame” in Table 1). Physicians sometimes delegated assessment of dementia severity to nurses. The participating physicians also completed a questionnaire about personal, non-patient related characteristics midway through the study. We used the data of the physician who provided end-of-life care. The local coordinating physician of each of 17 physician teams of 17 long-term care organizations that covered the 28 facilities completed a questionnaire on facility characteristics at the start, midway through, and at the conclusion of the study.

### Setting

Physicians were on the staff of the nursing home facilities and most were certified as elderly care physicians after a three-year vocational training [39]. Spiritual counselors were available and employed through the 17 long-term care organizations. Dutch long-term care facilities are required to offer spiritual care, and Bachelor or Master-level trained and certified spiritual counselors are available serving all denominations [40]. Formal spiritual care such as rituals by clergy from the community or visits by spiritual counselors on the staff was coordinated by physicians or arranged by families.

### Outcome measure

The outcome was spiritual care provision “shortly before death” as perceived by the on-staff elderly care physician. For this, we combined the response options provided to the physician of “spiritual care provided involving the last sacraments, or another last rite,” “no last rites but spiritual care was provided to patient by a spiritual counselor,” and “no last rites but spiritual care was provided to the patient by nursing home staff not specialized in spiritual care.” Referring to last rites, we also asked how many days before death these were administered.

### Potential predictors

For hypotheses driven rather than data driven analyses, we searched for potential predictors in previous work and in the comprehensive DEOLD dataset in an iterative way and found that most referred to one or more of three concepts: (1) a higher quality of care overall, and more specifically, (2) a more individualised or more person-centered approach, including considering religious backgrounds, and (3) a palliative care approach, and further, (4) possibly also to factors such as demographics. These concepts are related and overlap; for example, palliative care is person-centered by definition as it addresses the specific needs of individual patients and families [15] but person-centered care is not necessarily palliative care. Table 1 lists the potential predictors at the level of facilities, physicians, and residents and their families along with variable definitions of items including timing and responses, and how they may be indicators of the three concepts above. Further, some potential predictors can indicate more concepts, for example, facilities with a strong religious affiliation may apply palliative care principles more consistently, and the affiliation has also been associated with more spiritual caregiving at the end of life in nursing homes [30] and more comfort in patients dying with dementia in long-term care facilities [31]. However, it may also attract a specific group of patients which may result in increased chances of spiritual wishes being met [9]. Further, urban areas may be more secularized, less familiar with last rites [14] and also provide lower quality of care (Table 1). Finally, religious background related to an individualised approach rather than demographics because we referred to concordance of religious backgrounds as an indicator of an individualised approach if unrelated to the provision of spiritual end-of-life care. We classified these indicators that may relate to more concepts with the concept for which we felt the association was most likely, and preferably with the more specific concept (e.g., palliative care over quality of care). We anticipated that some factors would be associated with outcome in univariable analyses only, such as demographics as perhaps related to the stronger predictor of religious background.

We examined potential predictors of spiritual end-of-life care at the level of the resident and family―using the family caregiver’s and physician’s baseline assessment, at the physician level, and the facility level. At the latter level, for variables assessed multiple times such as enough nurse staffing, we selected the last assessment rather than the first, because the facility characteristics proximate to the time when most residents died are most likely to affect the outcome (i.e. the opposite direction, the patient-level outcome affecting facility characteristics is unlikely). With regard to communication variables with both a physician and a family perspectives, we opted for the family perspective for reasons of relevance and to avoid using the same perspective for predictor and outcome (assessed by the physician). As regards the quality of communication or the relationship with either the physician or the nurses, we selected the variables referring to the physicians as the focus of our work.

### Selection of residents

We included 372 newly admitted residents, and 218 residents died (59%) during the assessment period. A complete physician’s after-death assessment was available for 213 residents [17]. We excluded 6 residents with missing outcome, resulting in 207 cases for analyses. The 88 physicians completed after-death assessments for 1 to 9 residents.

### Analyses

We performed Generalized Estimating Equations (GEE) regression analyses to adjust for clustering with physicians and facilities (resident- and physician-level variables, and multivariable analyses) or facilities only. Associations of independent variables as defined in Table 1 were determined with the provision of spiritual end-of-life care as the dependent variable. We calculated confidence intervals (95% CI). From each of the three concepts (1–3), out of the four to six factors each operationalized with one or more variables in Table 1, based on the Wald chi-square, we selected for (theory-driven) multivariable analyses the variable with the strongest association with the outcome in univariable analyses. From the category of other factors (4), we included all factors that were significant in univariable analyses. We also tested a (statistics-driven) model that included all variables that were significant in univariable analyses, regardless of the concept it may refer to. All analyses were performed with SPSS 20.0.0 (IBM, 2011).

### Missing data and death before the baseline assessment

Some missing data were due to residents moving to other, non-participating facilities (6/7 who moved), in which case we invited the attending physician of the new facility to complete the resident-level assessments and the physician assessment. We then assessed only publicly available facility characteristics (e.g., number of beds). Missing physician-level items were mostly due to physicians changing employment status early. Other possibly non-at random missing data were mainly due to residents dying before the baseline assessment, before physicians and families had had a chance to complete it prospectively. For these residents we used shortened baseline assessments to retrospectively collect only the data deemed not particularly vulnerable to recall bias.

In 37 cases either the physician assessment (10; 8 with regular family caregiver assessments, 2 lacking the family caregiver baseline assessment) or the family caregiver assessment (4; with regular physician assessments) or both (22) were only performed after death, and in one case the physician completed the baseline questionnaire almost nine months later yet still before death. We examined whether the outcome and potential predictor variables differed between the 24 (2 + 22) cases completely lacking prospective data and the other 183 cases.

Missing data were imputed with the multiple imputation procedure implemented in SPSS. For multiple imputation in multivariable analyses, we used the information of all full, single, variables with significant associations in univariable analyses; for variables related to religion, faith or spirituality, we selected the strongest of either resident or family to avoid collinearity and redundancy. We customized for 15 imputed datasets, and a maximum of 50 iterations, and the “predictive mean matching” option to avoid out-of-range imputations. We calculated ORs and 95% CIs from the summary coefficients and SE. For comparison, we also ran the multivariable model with simple imputation of mode and mean scores. To check for possible differences due to selective missing, we repeated the procedure limiting to the 183 cases for which prospectively collected data were available, and also examined addition of, and interaction with, a variable that adjusted for this in analyses of the full dataset.

## Results

Table 2 and the left columns of Tables 3, 4, 5 and 6 describe the facilities, physicians, residents and their families grouped by the concept they may refer to. The facility size was variable (11–210 beds) and a minority were residential homes, had a religious affiliation or were urban (Table 2). Less than half (10/28) had small-scale living arrangements or a palliative care unit, and the coordinating physician considered nurse staffing insufficient for half of facilities. Facility characteristics weighted for number of residents (Tables 3, 4, 5 and 6) were largely similar, except for small-scale living arrangements, which in some facilities were available for only some of the residents.

**Table 2:** **Characteristics of the facilities in which the selected 207 residents resided including after having moved to other facilities**

Numbers refer to number of facilities unless indicated otherwise	Facility of:	
	Admission (n = 28)	Death (n = 34)
Nursing home	23	29
Residential home	5	5
Strong religious affiliation	3	3
No religious affiliation or only historically	25	31
Located in town	23	27
Located in large city	5	7
Staffing: enough nursing staff	14	-*
Staffing: not enough	14	
Palliative care unit	10	-*
No palliative care unit	18	
Small-scale living for dementia: all residents	5	-*
Small-scale living for dementia: some of the residents	5	
No small-scale living for dementia available	18	
Facility size – number of psychogeriatric (dementia) care beds, range	11-210	11-210

^*^Data not available from the 6 non-participating facilities to which 6 of 7 residents moved.

**Table 3:** **Univariable associations of the provision of spiritual end-of-life care as perceived by physicians with potential predictors related to quality of care**

	Descriptives	Spiritual care at the end of life^‡^		Association with the provision of spiritual care; OR (95% CI)
		Provided	Not provided	***Significant associations are italicized and bolded***
*Facility level* ^*^				
Nursing home vs. residential home, %	92.3	83.7	94.5	0.32 (0.08; 1.2)
Located in town versus large city, %	19.8	14.0	21.3	0.33 (0.09; 1.2)
Enough nursing staff, %	50.0	52.4	49.4	1.4 (0.48; 4.0)
*Resident level*				
Satisfaction with care (mean EOLD-SWC score, SD)	30.3 (4.2)	31.7 (3.9)	29.9 (4.2)	***1.10 (1.01; 1.21)***
Satisfied with communication with the physician				
- Mean 0–3 scale (SD)^†^	1.7 (1.0)	2.1 (0.88)	1.6 (1.1)	***1.6 (1.1; 2.3)*** per point increment
- Percentage				
- Not satisfied	7.1	2.9	8.1	
- No talk but had wanted to	10.6	5.9	11.8	
- Neutral	18.8	5.9	22.1	
- No talk but accepted	1.8	0	2.2	
- Satisfied about the main elements	36.5	50.0	33.1	
- Satisfied in every respect	25.3	35.3	22.8	

EOLD-SWC = End-Of-Life care in Dementia–Satisfaction With Care; range 10–40 with higher scores representing more satisfaction.

^*^Facility characteristics refer to the facility where resident died (34 facilities; in 200 cases, same as facility of admission; in 7 cases, other facility) and descriptives are weighted for number of residents who died in the facility.

^†^In 0–3 scale, combined “no talk but had wanted to” with “not satisfied” and “no talk but accepted” with “neutral”.

^‡^For dichotomous variables, the proportion for which spiritual care was provided and not provided can be calculated as well reconstructing the 2x2 table and taking into account possible missing values as listed in Table 1. For example, 0.923 * 207 (no missing values) = 191 resided in nursing homes, so 207 – 191 = 16 in residential homes. Of those for whom spiritual end-of-life care was provided (43), 0.837*43 = 36 resided in nursing homes, so 7 in residential homes. The proportions (percentages) who were provided spiritual care at the end of life, were therefore 36/191 (18.8%) life in nursing homes, and 7/16 (43.8%) in residential homes.

**Table 4:** **Univariable associations of the provision of spiritual end-of-life care as perceived by physicians with potential predictors related to individualised, person-centered care and religiousness variables**

	Descriptives	Spiritual care at the end of life^†^		Association with the provision of spiritual care; OR (95% CI)
		Provided	Not provided	***Significant associations are italicized and bolded***
*Facility level* ^*^				
Small-scale living, % (at resident level)	18.0	14.3	18.9	0.78 (0.27; 2.3)
Strong religious affiliation,%	9.2	30.2	3.7	***9.9 (1.6; 62)***
*Physician level*				
Any specific religious background physician, %	61.3	74.4	57.8	1.9 (0.73; 5.0)
Importance of faith or spirituality physician, %				
- Not at all important	13.4	10.3	14.3	***2.7 (1.1; 7.0)*** (very important versus other)
- Somewhat important	48.4	38.5	51.0	
- very important	31.2	48.7	26.5	
- Don’t know	7.0	2.6	8.2	
Frequency of attending religious services physician				
- Mean 0–5 scale (SD)	1.2 (1.6)	2.2 (1.9)	0.9 (1.4)	***1.6 (1.2; 2.1)*** (per 1-point increment)
- Percentage				
- Never	53.2	30.8	59.2	
- Once or twice a year	14.5	15.4	14.3	
- Once a month or so	11.3	12.8	10.9	
- Two or three times a month	7.5	5.1	8.2	
- Every week	8.6	20.5	5.4	
- More than once a week	4.8	15.4	2.0	
*Resident level*				
Any specific religious background resident, %	76.9	97.4	72.0	***13 (1.6; 103)***
Any specific religious background, %				
- Both resident and physician	49.1	74.3	42.9	***17 (2.1; 131)***
- Resident only	25.7	22.9	26.4	8.6 (0.92;80)
- Physician only	12.6	0	15.7	Reference
- Neither	12.6	2.9	15.0	Reference^‡^
Importance of faith or spirituality resident, %				
- Not at all important	34.9	5.3	42.0	***12 (5.1; 28)*** (very important versus other)
- Somewhat important	30.8	18.4	33.8	
- Very important	31.3	76.3	20.4	
- Don’t know	3.1	0	3.8	
Faith or spirituality very important, %				
- Both resident and physician	14.3	40.0	7.9	***21 (6.1; 74)*** ^***¶***^
- Resident only	16.0	37.1	10.7	***15 (5.2; 44)*** ^***¶***^
- Physician only	17.1	8.6	19.3	2.0 (0.44; 9.1)
- Neither	52.6	14.3	62.1	Reference
Frequency of attending religious services resident				
- Mean 0–5 scale, SD	2.0 (2.0)	3.6 (1.6)	1.6 (1.8)	***1.8 (1.4; 2.2)***/1-point increment
- Percentage				
- Never	37.9	5.3	45.9	
- Once or twice a year	14.4	15.8	14.0	
- Once a month or so	2.6	0	3.2	
- Two or three times a month	9.2	7.9	9.6	
- Every week	23.1	34.2	20.4	
- More than once a week	12.3	36.8	6.4	
- Don’t know	0.5	0	0.6	
Any specific religious background family, %	63.9	89.5	57.7	***5.5 (2.2; 14)***
Importance of faith or spirituality family, %				
- Not at all important	36.1	7.9	42.9	***4.5 (2.1; 9.9)*** (very important versus other)
- Somewhat important	39.2	44.7	37.8	
- Very important	21.6	47.4	15.4	
- Don’t know	3.1	0	3.8	
Frequency of attending religious services family				
- Mean 0–5 scale, SD	1.1 (1.6)	2.6 (1.9)	0.79 (1.2)	***1.9 (1.5; 2.3)***/1-point increment
- Percentage				
- Never	50.3	18.4	58.1	
- Once or twice a year	24.4	23.7	24.5	
- Once a month or so	5.7	7.9	5.2	
- Two or three times a month	6.7	10.5	5.8	
- Every week	6.7	13.2	5.2	
- More than once a week	6.2	26.3	1.3	
- Don’t know	0	0	0	
Family trust in physician				
- Mean 1–5 scale (SD)	4.04 (0.61)	4.12 (0.54)	4.01 (0.62)	1.3 (0.67; 2.3)/1-point increment
- Percentage				
- Very little	0	0	0	
- Little	0.6	0	0.7	
- Somewhat	14.7	8.8	16.2	
- A great deal (large amount)	65.3	70.6	64.0	
- A very large amount	19.4	20.6	19.1	

^*^Facility characteristics refer to the facility where resident died (34 facilities; in 200 cases, same as facility of admission; in 7 cases, other facility) and descriptives are weighted for number of residents who died in the facility. Small-scale living represent resident-level analyses.

^†^The footnote to Table 3 provides an example of how to reverse column and row percentages of dichotomous variables to result in proportions of residents who were provided spiritual end-of-life care with each of two response options.

^‡^Estimates do not converge with the last category only as the reference; we therefore combined with the before-last category.

^¶^p = 0.558 for difference between upper two options.

**Table 5:** **Univariable associations of the provision of spiritual end-of-life care as perceived by physicians with potential predictors related to palliative care**

	Descriptives	Spiritual care at the end of life^†^		Association with the provision of spiritual care; OR (95% CI)
		Provided	Not provided	***Significant associations are italicized and bolded***
*Facility level* ^*^				
Palliative care unit, %	38.5	38.1	38.6	0.72 (0.17; 3.1)
*Resident level*				
Comfort goal of care, %	62.4	60.6	62.8	0.77 (0.34; 1.7)
Family expected death one month before, %				
- Yes	33.0	35.9	32.1	1.2 (0.67; 2.3) (expected versus other)
- No	59.7	56.4	60.6	
- Don’t know	7.4	7.7	7.3	
Perception of dementia as a disease you can die from	3.4 (1.2)	3.1 (1.2)	3.5 (1.2)	0.82 (0.57; 1.2)/per 1-point increment agreement
- Mean 1–5 scale				
- Percentage				
- Completely disagree	9.5	14.7	8.1	
- Partly disagree	8.3	8.8	8.1	
- Neither agree, nor disagree	13.6	23.5	11.1	
- Partly agree	14.2	11.8	14.8	
- Completely agree	26.6	17.6	28.9	
- Don’t know	27.8	23.5	28.9	

^*^For facility level, descriptives are weighted for number of residents who died in the facility.

^†^The footnote to Table 3 provides an example of how to reverse column and row percentages of dichotomous variables to result in proportions of residents who were provided spiritual end-of-life care with each of two response options.

**Table 6:** **Univariable associations of the provision of spiritual end-of-life care as perceived by physicians with other potential predictors including demographics**

	Descriptives	Spiritual care at the end of life^†^		Association with the provision of spiritual care; OR (95% CI)
		Provided	Not provided	***Significant associations are italicized and bolded***
*Facility level* ^*^				
Facility size, number of psychogeriatric (dementia) care beds	110 (SD 51)	96 (58)	113 (58)	***0.991 (0.982; 1.000*** ***)*** ^*‡*^/bed
*Physician level*				
Female gender physician, %	62.6	65.9	61.7	1.1 (0.46; 2.8)
Age physician (resident level), mean number of years (SD)	43.1 (8.7)	42.1 (9.1)	43.4 (8.7)	1.00 (0.95; 1.05) per year
*Resident level*				
Female gender resident, %	66.2	67.4	65.9	1.2 (0.52; 2.6)
Age resident, mean (SD)	85.3 (6.4)	86.4 (5.9)	85.1 (6.5)	1.03 (0.97; 1.09) per year increment
Dementia severity, mean BANS-S score (SD)	14.6 (4.5)	15.3 (4.3)	14.4 (4.5)	1.03 (0.96; 1.11) per point increment
Female gender family, %	61.5	72.1	58.6	***2.4 (1.1; 5.1)***
Age family, mean (SD)	61.1 (11.7)	58.6 (10.2)	61.7 (12.0)	0.97 (0.94; 1.01) per year increment
Relationship family with resident, %				
- Spouse or partner	20.0	15.4	21.2	Reference
- Child	59.5	66.7	57.7	1.5 (0.59; 4.0)
- Other	20.5	17.9	21.2	1.3 (0.43; 3.8)

^*^For facility level, descriptives are weighted for number of residents who died in the facility.

^†^The footnote to Table 3 provides an example of how to reverse column and row percentages of dichotomous variables to result in proportions of residents who were provided spiritual end-of-life care with each of two response options.

^‡^p = 0.046.

The residents and family caregivers were mostly female; mean ages were 85.3 and 61.1 years respectively (Table 6). Most physicians had a specific religious background (61.3%; Table 4), yet less than one-third of them considered faith or spirituality very important (31.2%), and most (53.2%) never attended religious services. The residents also frequently had a specific religious background (76.9%; Table 4), but in pairwise comparisons, in a quarter of cases (25.7%) only the resident had any such background where the physician had only in 12.6% of cases. Similar to the physicians, less than one-third of the residents (31.3%) found faith or spirituality very important, but the majority (62%) had attended religious services. Fewer families reported faith or spirituality being very important (21.6%), and on religious background (63.9%) or never attending services (50.3%) they were closer to the physicians than to the residents (Table 4). In 80.4% of cases, the resident having or not having a religious background corresponded with the family (not in Table).

Regarding quality of care at baseline (Table 3), only one-quarter of families (25.0%) were satisfied with the communication with the physician in every respect. Over one in ten (12.4%) had not yet talked to the physician at 8 weeks from admission, and this was dissatisfying to most (18/21 of those who had not yet talked to the physician; overall 10.6%, and 1.8% who accepted it, Table 3). Regarding palliative care (Table 5), the physicians reported a comfort goal of care at baseline for most residents (62.4%). Only one-third of families (33.0%) reported having expected death the month before.

### Spiritual end-of-life care

According to the physicians, spiritual end-of-life care was provided shortly before death to a total of 20.8% (43/207) of residents. This involved pastoral care with last sacraments, or another last rite (overall 8.2%; 40%, 17/43 of those for whom spiritual end-of-life care was provided), provided on average 2.5 days before death (SD 2.0, range 0–8 days). Further, a spiritual counselor provided other types of spiritual care for 11.1% of residents (53%, 23/43 of those for whom spiritual end-of-life care was provided) and a staff member not specialized in spiritual care did so in 1.4% of cases (7%, 3/43 of those for whom spiritual end-of-life care was provided). The 24 residents for whom prospective data was lacking completely were equally likely to receive spiritual end-of-life care (25.0% versus 20.2% in other 183 cases; p = 0.56).

### Single potential predictors of spiritual end-of-life care

In univariable analyses, families’ baseline satisfaction with care, and satisfaction with physician communication predicted the provision of spiritual end-of-life care (Table 3). Further, residents of facilities with a strong religious affiliation were more likely to receive spiritual end-of-life care as perceived by the physicians (Table 4). The physician’s religious background was unrelated, but residents whose attending physicians found faith or spirituality very important in life, or who attended religious services more frequently, were more likely to receive such care. Similarly, the importance attached to faith or spirituality and attending services by resident and family predicted the provision of spiritual end-of-life care, but having any such background was also predictive.

The physician as well as the resident having a spiritual background (compared to physician only or neither) predicted receipt of spiritual end-of-life care (OR 17, CI 2.1;131), but the association was less strong and not significant if the resident had such a background and the physician did not (OR 8.6; CI 0.92;80). The main predictor of the importance of faith or spirituality item in relation to spiritual end-of-life care was the *resident* finding it important, irrespective of the physician attaching importance to it (p = 0.56 for “resident only” when reference reversed to upper category “both resident and physician”). Trust was not significantly associated with the outcome.

Further, none of the indicators of palliative care was significantly associated with the outcome (Table 5). Of the other factors, a smaller number of dementia care beds was predictive, in addition to family caregivers being female (Table 6), also when adjusted for the three variables indicating religion, spirituality or faith.

Of all items listed in Tables 3, 4, 5 and 6, only a few resident-level variables differed for the 24 residents who died soon after admission: on average, they were younger and had more severe dementia. The adjustment, however, did not change the ORs for age and dementia severity (remained 1.03 per year or point increment for both).

### Independent predictors of spiritual end-of-life care

Independent predictors of the provision of spiritual end-of-life care when including the strongest predictor among the indicators of each of the concepts (the theory-driven model; Table 7) were resident-level factors: families’ satisfaction with physician communication at baseline (OR 1.6, CI 1.0;2.5 per point on the 0–3 scale) and faith or spirituality very important to resident regardless of importance to the physician (OR 19, CI 5.6;63 and OR 15, CI, 5.1;47, respectively) versus not important for both. As in univariable analyses, none of the indicators for a palliative care approach was predictive. Further, residents with a female family caregiver at baseline were more likely to receive spiritual end-of-life care (OR 2.7, CI 1.1;6.6).

**Table 7:** **Independent predictors of the provision of spiritual end-of-life care as perceived by physicians (n = 207, multivariable analyses with multiple imputation)**

	Independent association with the provision of spiritual end-of-life care; OR (95% CI)
	***Significant associations are italicized and bolded***
**(1)** ^*^ Satisfied with communication with the physician, 0–3 scale	***1.6 (1.04; 2.5) per point increment (p = 0.034)***
**(2)** Faith or spirituality very important	
- Both resident and physician	***19 (5.6; 63)***
- Resident only	***15 (5.1; 47)***
- Physician only	2.2 (0.46; 10)
- Neither	Reference
**(3)** Family expected death one month before	1.3 (0.51; 3.3)
**(4)** (a) Facility size, number of psychogeriatric (dementia) care beds	0.997 (0.987; 1.007)/bed
(b) Female gender family	***2.7 (1.1; 6.6)***

^*^The numbers between brackets refer to the categories as listed in Table 1 and univariable analyses presented in Tables 3, 4, 5 and 6: (1) Quality of care, (2) A more individualised or more person-centered approach of care; religious backgrounds, (3) Palliative care, (4) Other factors or unclear expectation with regard to the direction of a possible association.

When limiting to cases with prospective data (183/207), families’ female gender was not a significant predictor, and the OR was somewhat smaller (2.1 vs. 2.7), also when compared to the full dataset with simple imputation (2.4, CI 1.2-4.8), but the results were similar when adjusted for missing prospective data and there was no significant interaction with gender. Analyses without imputation, and analyses including all the variables significant in the univariable analyses (the statistics-driven model) resulted in the same three variables being significantly associated with the outcome and no additional significant variables.

## Discussion

In our prospective study in Dutch long-term care, we found that independent predictors of the provision of spiritual end-of-life care in dementia as perceived by their physicians included families’ reports of satisfaction with physicians’ communication soon after admission (at baseline), and families’ reports of faith or spirituality having been very important to the resident irrespective of the importance to the physician. These findings were robust to restriction of samples, imputation methods and theory-driven or statistics-driven regression methods. We also found that female caregiving was independently associated with spiritual end-of-life care but only when cases of death soon after admission were included.

Early established good family-physician communication may increase chances of spiritual caregiving. A retrospective study found that US residents of VA long-term care facilities were more likely to be visited by a chaplain if a family member was involved at the end of life [41]. In our study, we also found that the caregiving was person-centered in the sense that physicians for whom faith or spirituality was unimportant for themselves also coordinated spiritual caregiving for the resident for whom this had been important.

Palliative care indicators as defined in our study were unrelated to spiritual end-of-life care, while spiritual care is explicitly included in definitions of palliative care [5, 15]. We may have lacked good indicators for palliative care, and such clear indicators may be needed and relevant beyond the particular study design. Dutch long-term care practice employs spiritual counselors, but the physicians may not have a clear view of what palliative care in dementia entails. There are no multidisciplinary specialist palliative care teams that explicitly support end-of-life caregiving as there are in Flanders [42], or the US (e.g., Li, et al. [43]) where hospice was associated with more frequent provision of spiritual end-of-life care in a retrospective study in long-term care settings [30]. Moreover, a Dutch interview study showed that elderly care physicians employed variable definitions, and some emphasized withholding treatment rather than providing treatment for comfort [44]. Some also felt that all care for nursing home residents with dementia is palliative, rendering it an indiscriminative indicator for the setting. Further, few knew the definitions of care goals issued by the professional organization [33]. Moreover, unlike the WHO definition of palliative care and a recent definition of palliative care in dementia specifically [5], the palliative care goal definitions of the Dutch professional organization do not refer explicitly to spiritual caregiving, as they were developed to discriminate a palliative care goal from life-prolongation as a goal [33].

In our study about one in five (20.8%) residents received spiritual end-of-life care, which is much lower than in a US four-state study asking bereaved family on spiritual caregiving in the last month of life (72.4%; [30]). It may be, however, an underestimation for the Netherlands, because the percentage was higher (47%, and mostly (38%) rituals) in the two organizations that we excluded from the analyses because they collected the data only retrospectively. These were situated in regions with a dominant Roman Catholic tradition. The predictors of spiritual end-of-life care, however, are not necessarily different in those areas.

Our outcome purposefully referred to spiritual care as perceived by the physician, suggesting more formal, religion-related and “visible” care in addition to any spiritual care provided by specialised and non-specialised staff. The last was provided in only 1.4% of cases. Nurses provide spiritual end-of-life care that is not formalized in care plans and is perhaps not documented either, as observed in Dutch ethnographic work [45]. Such spiritual caregiving may not have been noted by the physicians, but is covered in models of spirituality in the literature [11, 46]. Predictors for such an informal spirituality-focused rather than religiousness-focused outcome may be dissimilar; for example, such care may be brought about and affected by other factors than physician communication and importance of faith or spirituality.

Our work may be relevant for other countries. For example, physician presence may be important for communication as both US and Dutch physician’s presence related to knowing family wishes better [18]. Further, it may be relevant to systems where not the physician, but another professional may have the role of coordinating the care which may include spiritual care.

Limitations of our study include the operationalization of spiritual caregiving and the three related concepts we examined. For example, we could not cover the full concept of person-centered care [47] and we had the fewest variables for palliative care, one of which (death expected) was assessed only after death. Some of these variables were not indicative in themselves, but their association with outcome was, such as the combined importance attached to faith or spirituality by both physician and resident. The rationale for this variable indicating person-centeredness is that spiritual care should not be provided to those who previously found this unimportant. This may be called in question and some may argue that spiritual care, which is not necessarily religious, should be provided to all, and especially at the end of life and with dementia, unless there is clear evidence of reluctance on the part of the patient.

To the best of our knowledge, this is the first study prospectively relating status shortly after admission to spiritual end-of-life care, and the first to focus on spiritual end-of-life care for residents of long-term care facilities as perceived by physicians. The retrospective US four-state study on correlates of spiritual care did not include physician perceptions but family perceptions, and while they also found that the importance of spirituality/religion to the resident was a strong correlate [30], there was no study of the importance to staff. A clear temporal relation reduces the risk of bias, but cautious interpretation as causal is still warranted because of the observational study design. Further, we did not follow all residents until death, so our sample selectively included more resident with a shorter length of stay, who were younger and had less advanced dementia, but there was no difference between the prospective and retrospective designs in important outcomes such as pain and comfort [17]. With 28 facilities and 88 physicians in the analytic sample, the power for facility-level and physician-level variables to detect associations with outcome was smaller than for resident-level variables.

The first assessment in DEOLD was eight weeks after admission to long-term care, and future work may focus on establishing physician-family contact in the first weeks. Such work may be qualitative or quantitative observational or experimental and provide more detailed clues as to how to facilitate (spiritual) caregiving at the end of life. We did not relate spiritual caregiving to patient outcomes such as quality of life or comfort because these were also assessed after death. In cancer patients, others found that being visited by a pastor at the end of life as well as being treated as a whole person and with respect, and trusting and respecting their physician predicted quality of life at the end of life [4].

## Conclusions

Physician or staff-family communication as early as within weeks from admission to a long-term care facility may be important in the provision of (spiritual) end-of-life care. Palliative care, in the absence of organizational structures indicating this care is provided, may need better defining and implementation in clinical nursing home practice and should explicitly include additional aspects of care such as spiritual end-of-life care.
